# Cardiac Myosin-Binding Protein C (cMyC) as a Novel Biomarker for Early Diagnosis of Myocardial Infarction: A Brief Review

**DOI:** 10.7759/cureus.94729

**Published:** 2025-10-16

**Authors:** Burak Akyuz

**Affiliations:** 1 Cardiology, Kırıkkale University Hospital, Kırıkkale, TUR

**Keywords:** biomarker, cardiac myosin-binding protein c, cardiac troponin, cmyc, early diagnosis and treatment, myocardial infarction

## Abstract

This article is a narrative review summarizing current evidence on the diagnostic value of cardiac myosin-binding protein C (cMyC) compared with conventional cardiac biomarkers. Cardiac myosin-binding protein C (cMyC) has emerged as a novel biomarker with potential advantages over conventional troponins for the early diagnosis of myocardial infarction (MI). Its rapid release and clearance kinetics may provide opportunities for improved risk stratification and patient management. This review explores the diagnostic potential of cMyC in the detection of MI and considers its possible role in post-infarction monitoring. A systematic literature search was conducted in PubMed, Embase, and the Cochrane Library from inception to September 2025 using the terms “cardiac myosin-binding protein C,” “cMyC,” “myocardial infarction,” “acute coronary syndrome,” and “biomarker.” Studies were included if they investigated cMyC kinetics, diagnostic accuracy, or its clinical application in patients with suspected or confirmed MI. Original clinical trials, observational studies, and translational research articles published in English were eligible. Review articles, editorials, case reports, and non-English publications were excluded. Evidence suggests that cMyC rises and falls more rapidly than high-sensitivity troponins, enabling earlier identification of MI and offering value for short-term monitoring following cardiac events. Compared to troponins, cMyC may improve early decision-making in the emergency setting. However, current research remains limited, and further studies are needed. cMyC is a promising biomarker for early MI diagnosis and hospital-based follow-up. Future large-scale, multicenter studies are essential to confirm its clinical utility and establish its role in existing diagnostic pathways.

## Introduction and background

Introduction

Cardiac myosin-binding protein C (cMyC) has recently attracted attention as a novel cardiac biomarker [1]. Its clinical potential has also been demonstrated in recent observational studies evaluating early diagnostic performance in acute coronary syndromes [2], and its diagnostic value has been further supported by comparative analyses in diverse clinical settings [3]. Early biochemical investigations also confirmed significant plasma elevations of cMyC following myocardial injury, reinforcing its diagnostic potential [4]. Its identification through proteomic analyses was first reported by Jacquet et al. [5]. Clinical translational studies have also demonstrated the early detectability of cMyC compared with high-sensitivity troponins, particularly in post-PCI myocardial injury settings [6]. Additionally, early discussions within the European Society of Cardiology (ESC) guidelines have mentioned cMyC in the context of emerging biomarkers [7].

The discovery of cMyC as a biomarker originated from proteomic investigations. Jacquet et al. (2009) identified cMyC through proteomic analysis of cardiac tissue and plasma, demonstrating that it is exclusively expressed in the myocardium and released into the circulation following infarction. This early work established the biological specificity of cMyC and provided the foundation for subsequent studies evaluating its diagnostic potential in clinical cohorts [5].

Cardiac myosin-binding protein C (cMyC) is a promising new biomarker for the early detection of myocardial infarction (MI). It enables rapid rule-in and rule-out of patients with suspected MI and may also be useful for monitoring patients who have undergone coronary angioplasty and are admitted to the cardiology ward [1-3].

Studies suggest that cMyC rises and falls more rapidly than other cardiac biomarkers, such as Troponin T and Troponin I, enabling earlier rule-out and rule-in of MI and potentially saving valuable time [4,6]. In contrast, troponin levels are released more slowly and often require repeated blood tests [1-3]. Because of this delayed release, patients with elevated troponin levels require repeated measurements, highlighting the need for newer markers that provide faster results in terms of resource use, time efficiency, and patient safety [1-3].

From a biological perspective, cMyC is encoded by the MYBPC3 gene and is expressed exclusively in the myocardium. It is more abundant than troponins within the sarcomere, which may partly explain its rapid appearance in the systemic circulation following myocardial injury. Recent studies have also established reference ranges and biological variation data for cMyC concentrations in healthy individuals [8,9]. Initially identified through proteomic analyses, cMyC has emerged as a highly specific and sensitive indicator of myocardial necrosis.

The clinical need for such a biomarker is substantial. Evidence from recent reviews and comparative studies [10] highlights ongoing diagnostic challenges. Emergency departments worldwide face an increasing number of chest pain presentations, as highlighted in recent ESC guideline discussions [7], with studies reporting that chest pain accounts for approximately 5-10% of all emergency visits [11]. The healthcare burden of acute chest pain has been widely discussed in previous research [10,11]. Current high-sensitivity troponin algorithms recommended in recent ESC guidelines [7] and further reviewed in contemporary literature [10] leave up to 30% of patients in an indeterminate “observation zone,” requiring repeat testing and prolonged monitoring before a definitive diagnosis can be made [12]. This contributes to longer emergency department stays, increased resource utilization, and higher workload for nursing and medical staff. Novel biomarkers such as cMyC that can accelerate triage and reduce this observation burden may streamline diagnostic pathways, improve patient flow, and optimize the use of emergency resources [4,7,10,12].

The first large multicenter validation study (Kaier et al., 2017) confirmed the feasibility of using cMyC in clinical cohorts, showing that it could safely classify approximately 10-15% more patients at baseline compared to troponin-based strategies [4].

Baker et al. (2015) provided important translational evidence by showing that cMyC was detectable significantly earlier than high-sensitivity troponin T in patients with iatrogenic MI following percutaneous coronary intervention, highlighting its potential as an ultra-early biomarker [6].

Current ESC and AHA/ACC guidelines recommend the use of high-sensitivity troponins within 0/1-hour or 0/2-hour diagnostic algorithms. The 2020 European Society of Cardiology (ESC) Non-ST-elevation (NSTE)-Acute Coronary Syndrome (ACS) guideline likewise bases its accelerated pathways on high-sensitivity troponins and has not yet incorporated cMyC pending further validation, highlighting the need for robust multicenter evidence before pathway integration [7].

This review summarizes findings from key studies to evaluate the diagnostic value of cMyC in MI and discusses its potential role as a complementary or alternative biomarker to existing algorithms.

## Review

Biomarkers in cardiology

Biomarkers are measurable biological indicators that reflect physiological or pathological processes and are widely used for diagnosis, prognosis, and therapeutic monitoring. In cardiology, biomarkers play an essential role in the early detection of MI, evaluation of heart failure, and risk stratification of acute coronary syndromes. An ideal cardiac biomarker should be specific to myocardial tissue, rise rapidly after injury, and correlate proportionally with the extent of damage.

Common cardiac biomarkers

The most established cardiac biomarkers include cardiac troponins (cTnT and cTnI), creatine kinase-MB (CK-MB), myoglobin, and B-type natriuretic peptide (BNP/NT-proBNP). Troponins are considered the gold standard for MI diagnosis due to their high sensitivity and specificity. However, they exhibit delayed release kinetics, often requiring serial testing within 3-6 hours. CK-MB and myoglobin rise earlier but lack myocardial specificity, leading to false positives in skeletal muscle injury. BNP and NT-proBNP are primarily used for diagnosing and prognosticating heart failure rather than acute infarction.

Emerging biomarkers and cMyC

Emerging biomarkers such as copeptin, heart-type fatty acid-binding protein (H-FABP), and cardiac myosin-binding protein C (cMyC) have been explored to overcome the limitations of troponin-based diagnosis. Among these, cMyC has shown the most consistent promise because it combines rapid release kinetics with high cardiac specificity. It is released earlier than troponins following cardiomyocyte necrosis and cleared faster from circulation, making it suitable for early rule-in/rule-out strategies. Studies suggest that cMyC improves early diagnostic accuracy, particularly in patients presenting within three hours of symptom onset [2-4,6]. Ongoing research aims to standardize assay platforms and integrate cMyC into future ESC diagnostic algorithms [7,8,10].

In a small single-center study, Panotopoulos et al. conducted a prospective analysis of 120 patients at Al-Yarmouk Teaching Hospital to evaluate the role of cMyC in the diagnosis of acute MI. The findings suggested that cMyC rises and falls more rapidly than troponins, indicating potential advantages for early diagnosis, although the small single-center design limits the generalizability of these findings [1].

The findings suggested that cMyC rises and falls more rapidly than troponins, indicating potential advantages for early diagnosis, although this conclusion is limited by the small single-center study design. Troponins are released more slowly and require repeated blood tests, and can also be elevated in conditions other than myocardial infarction [1].

Consequently, cMyC has been shown to be a potential biomarker for early diagnosis of ACS and for differentiation of its subtypes [1].

In a large-scale study conducted by Kaier et al., a total of 1,495 patients were evaluated, excluding those with renal failure, and 17% of them were diagnosed with NSTEMI. Patients with terminal renal failure and those receiving chronic dialysis were excluded from the study. The median age was 62 years, 31% of the participants were female, and 37% had a prior history of coronary artery disease [2].

The diagnostic performance of cMyC was compared with high-sensitivity troponin T and I. Algorithms were developed using blood samples taken at 0 and 1 hours. Rule-out criteria for cMyC were <10 ng/L or <18 ng/L at 0 hours, with a 0-1-hour increase of <4 ng/L. Rule-in criteria were ≥140 ng/L at 0 hours, or a 0-1-hour increase of ≥15 ng/L.

In a 663-person subcohort analysis, 49% of patients were classified as rule-out, 18.6% as rule-in, and 32.4% as observation. The negative predictive value (NPV) for the rule-out group was 99.5% and the sensitivity was 99.1%. The positive predictive value for the rule-in group was 70.6% [2].

The most striking result is that cMyC appeared to rule out or rule in more patients with only a single 0-hour measurement, although long-term validation studies are still required to confirm its safety and prognostic reliability. This offers a practical advantage compared to the existing troponin-based 0/1-hour algorithms of the ESC. cMyC has the potential to facilitate rapid diagnosis and improve patient management in the emergency department [2].

Govindan et al. analyzed plasma samples from both MI patients and healthy controls to assess the role of cardiac myosin-binding protein C (cMyBP-C) levels in MI diagnosis. Plasma cMyBP-C levels in MI patients were significantly higher compared to controls, increasing approximately 4.8-fold, with a rapid decline during post-MI follow-up. Receiver operating characteristic (ROC) analysis revealed that certain threshold levels provided high specificity and good sensitivity, although the relatively small cohort size restricts the strength of these conclusions [3].

The researchers concluded that cMyBP-C could be used as a sensitive and cardiac-specific biomarker in MI diagnosis, supporting its potential clinical use in both acute diagnosis and post-MI monitoring [3].

In a landmark multicenter cohort study (APACE; Kaier et al., 2017), nearly 2,000 patients presenting with suspected acute MI were evaluated for cMyC alongside high-sensitivity troponins [4]. The study found that cMyC achieved diagnostic performance comparable to hs-cTnT and hs-cTnI overall, but offered distinct advantages in patients presenting within three hours of symptom onset. Specifically, cMyC reduced the size of the indeterminate ‘observation zone’ by allowing more patients to be safely classified into either rule-out or rule-in groups on the basis of a single admission sample. Importantly, long-term prognostic assessment revealed that cMyC performed similarly to troponins in predicting mortality, further supporting its clinical relevance [4]. These findings highlight the potential of cMyC as a complementary biomarker that may expedite decision-making in the emergency department.

Baker et al. (2015) investigated the release of cMyC in patients with iatrogenic MI following percutaneous coronary intervention. Their study demonstrated that cMyC was detectable in the circulation significantly earlier than high-sensitivity troponin T. This early rise highlights the potential role of cMyC as an ultra-early biomarker, particularly in interventional settings where rapid recognition of myocardial injury is crucial. These findings provided translational evidence that cMyC could complement or even precede troponins in the detection of acute cardiac injury [6].

Recent work by Kloska et al. (2023) determined reference values and biological determinants for cMyC in a healthy adult population, establishing a 99th percentile upper reference limit of approximately 42.3 ng/mL. Female sex was associated with slightly higher concentrations, while age, BMI, and lipid profile showed no significant effects. These data are essential for assay calibration and cut-off selection in future clinical applications [8].

**Figure 1:**
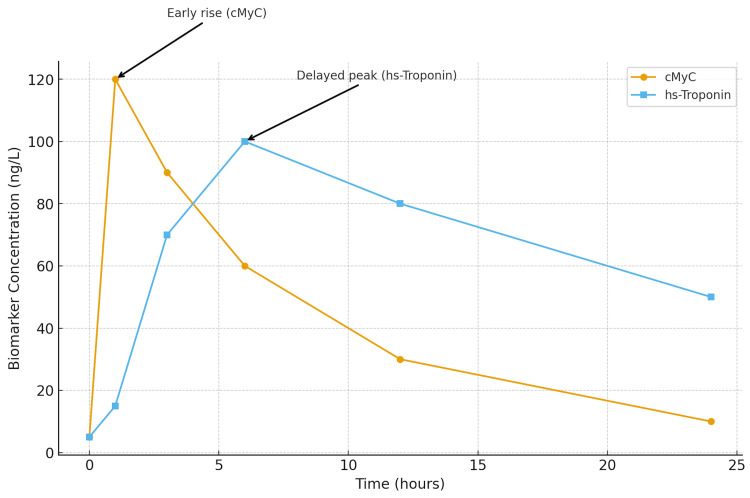
Time-concentration profiles of cardiac myosin-binding protein C (cMyC) and high-sensitivity troponin following myocardial infarction The schematic adaptation is based on Kaier et al., Circulation 2017; and Govindan et al., Am J Cardiovasc Dis 2013. cMyC: cardiac myosin-binding protein C; hs-Troponin: high-sensitivity troponin Schematic summary based on published data.

These kinetic differences are visually summarized in Figure 1, which schematically compares the time-concentration profiles of cMyC and high-sensitivity troponin following MI. The figure highlights the earlier rise and faster decline of cMyC compared to troponin, supporting its potential use in rapid rule-in/rule-out algorithms. The kinetic profile of cMyC compared with hs-Troponin is illustrated in Figure 1.

In addition, Alaour et al. (2021) investigated the weekly biological variation of cMyC in healthy volunteers, reporting a within-subject coefficient of variation of approximately 17.8% and a between-subject variation of 66.9%. The calculated reference change values and indices of individuality indicate that serial measurements of cMyC may provide meaningful information for patient monitoring [9].

In addition, a contemporary expert review by Marber et al. (2021) summarized translational and clinical evidence for cMyC, highlighting its myocardial specificity, rapid release kinetics, and potential role as a complementary biomarker to high-sensitivity troponins in acute care pathways [10].

**Figure 2:**
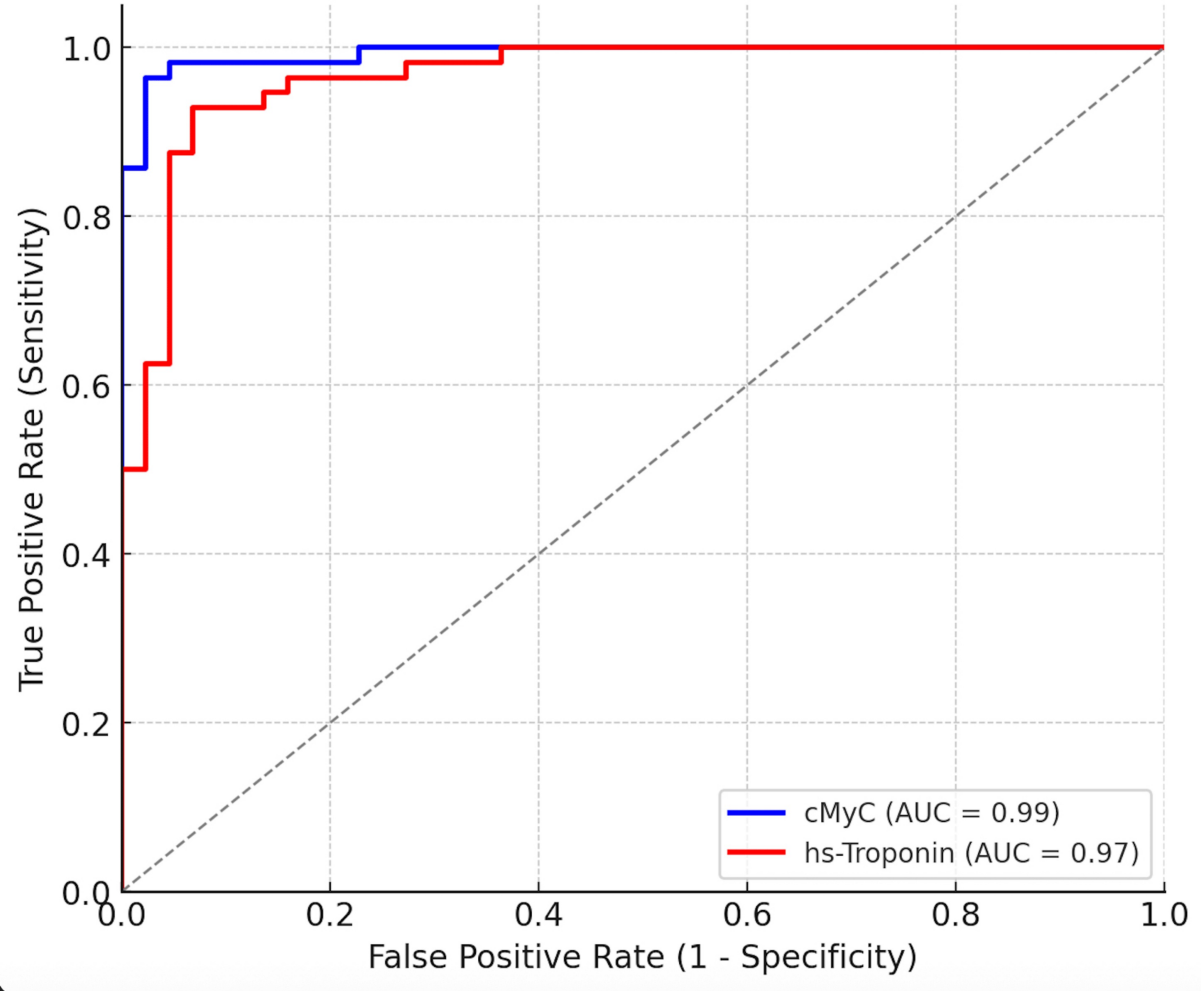
Comparative receiver operating characteristic (ROC) trends for cMyC and high-sensitivity troponin in myocardial infarction diagnosis. Schematic adaptation based on Kaier TE et al., Circulation 2017; and Panotopoulos C et al., J Adv Med Med Res 2022). ROC curve: Plot of sensitivity vs 1-specificity; AUC: Area under the ROC curve, a measure of overall diagnostic accuracy; cMyC: Cardiac myosin-binding protein C; hs-Troponin: High-sensitivity troponin This figure is a conceptual summary illustrating previously reported diagnostic performance trends. It does not represent original or newly generated data.

The comparative diagnostic performance between cMyC and high-sensitivity troponins across published studies is conceptually summarized in Figure 2. This schematic illustrates typical ROC trends, highlighting the slightly larger area under the curve (AUC) for cMyC reported in early analyses, particularly among patients presenting within the first few hours after symptom onset. It visually reinforces cMyC’s potential to improve early diagnostic discrimination when used alongside conventional troponins. A comparison of ROC curves for cMyC and hs-troponin is illustrated in Figure 2, demonstrating the potential diagnostic advantage of cMyC.

Discussion

Initial studies have highlighted the promising value of cMyC as a standalone cardiac biomarker for MI diagnosis. Its rapid rise and fall compared to troponins may provide advantages for early rule-in/rule-out and for monitoring patients following cardiac intervention, although these findings should be interpreted cautiously given the methodological limitations and small sample sizes of the cited studies [1-4]. This aligns with the conclusions of Marber et al. (2021), who emphasized the translational potential of cMyC and its complementary role alongside established troponins [10].

Kaier et al., with a large patient cohort, demonstrated that even a single baseline measurement of cMyC can safely classify a greater proportion of patients, making it particularly relevant for clinical practice [2]. Panotopoulos et al., despite a smaller sample size, emphasized the benefit of cMyC’s faster kinetics compared with troponin, supporting its use in early MI detection [3]. Govindan et al. further demonstrated that cMyC levels not only rise acutely but also decline rapidly post-MI, suggesting utility for in-hospital follow-up; inclusion of a healthy control group strengthened the findings [3].

In addition, Baker et al. (2015) provided important translational evidence by analyzing cMyC release in patients with iatrogenic MI following percutaneous coronary intervention. Their study demonstrated that cMyC appeared in the circulation significantly earlier than high-sensitivity troponin T, reinforcing the kinetic advantage suggested by earlier experimental data. This finding highlights the potential utility of cMyC as an ultra-early biomarker in interventional cardiology, where rapid recognition of myocardial injury is essential [6].

However, substantial limitations affect the interpretation of current evidence. cMyC has not consistently shown a major advantage over troponins in the 0/1-hour algorithm, and its clinical performance beyond short-term rule-in/rule-out strategies requires further validation. Smaller sample sizes in Panotopoulos and Govindan’s studies, as well as the exclusion of patients with advanced renal disease in Kaier’s cohort, restrict generalizability [3]. Long-term outcomes related to cMyC-guided management have yet to be investigated.

An additional consideration concerns the lack of standardization among cMyC assay platforms. Different analytical methods - including enzyme-linked immunosorbent assays (ELISA), immunoassays, and prototype high-sensitivity assays - have been used across studies, each with varying detection limits and calibration standards [3,4,8,9]. This inter-assay variability complicates direct comparison of reported concentrations and diagnostic thresholds. For instance, Kloska et al. (2023) established reference values using one commercial ELISA platform, whereas Kaier et al. (2017) employed a research-grade assay with distinct sensitivity characteristics, resulting in differing upper reference limits [4,8]. The absence of harmonized calibration procedures currently represents a major barrier to clinical translation and may contribute to inconsistencies in reported diagnostic performance across studies.

Overall, cMyC shows potential as an alternative or complementary biomarker to the ESC 0/1-hour troponin algorithm [4]. Additional evidence highlights its rapid post-MI decline and potential role in patient monitoring [7]. Expert reviews further emphasize its clinical utility as a complementary marker [10]. Larger, multicenter studies, ideally incorporating combined use with troponins, are warranted to define its role in clinical practice [1-3].

The results of Kaier et al. (2017) provide pivotal evidence for the clinical utility of cMyC in the early diagnosis of acute MI. In their large multicenter APACE cohort, cMyC demonstrated diagnostic accuracy comparable to high-sensitivity troponins overall, while offering unique advantages in patients presenting within three hours of symptom onset [4]. Notably, cMyC reduced the size of the indeterminate observation zone by safely reclassifying more patients into either rule-out or rule-in groups on the basis of a single admission sample. This capacity to streamline triage has significant implications for improving patient flow and reducing emergency department congestion. Furthermore, long-term prognostic assessment showed that cMyC performed similarly to troponins in predicting mortality, underscoring its potential as both a diagnostic and prognostic biomarker. Despite these promising findings, external validation and prospective trials remain necessary before cMyC can be recommended for routine integration into clinical practice guidelines.

**Figure 3:**
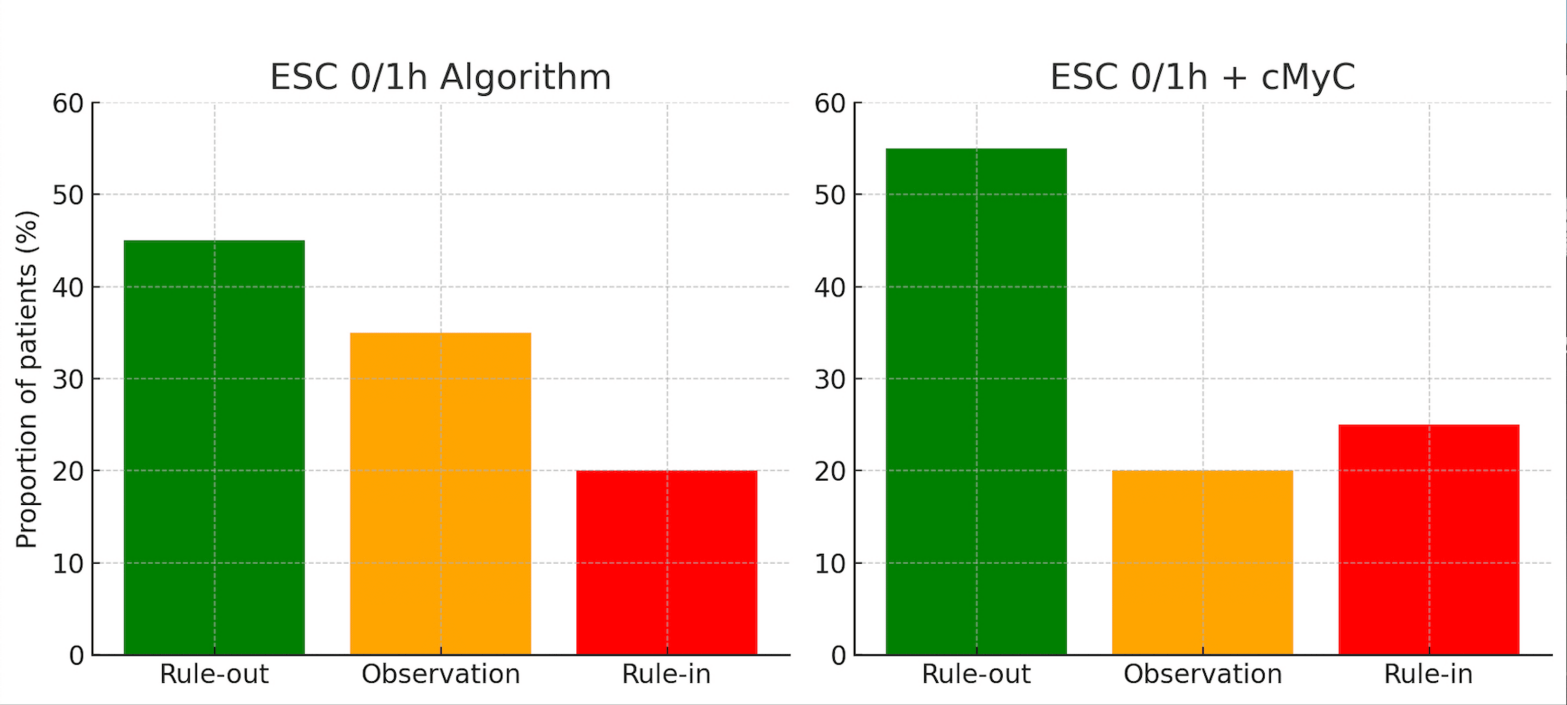
Comparison of patient classification using the ESC 0/1h algorithm alone versus in combination with cMyC Schematic adaptation based on Kaier TE et al., Circulation 2017. Green (Rule-out): Patients safely excluded from myocardial infarction at presentation, Orange (Observation): Patients in the indeterminate zone requiring repeat testing, Red (Rule-in): Patients with findings strongly suggestive of myocardial infarction ESC 0/1h algorithm: Standard European Society of Cardiology accelerated diagnostic pathway using high-sensitivity troponins cMyC incorporation: Addition of cardiac myosin-binding protein C at baseline to reduce the observation zone This figure provides a visual summary of published data trends and is not based on new analysis.

The potential clinical impact of incorporating cMyC into existing diagnostic algorithms is depicted in Figure 3. This schematic contrasts the standard ESC 0/1-hour high-sensitivity troponin algorithm with a combined approach including cMyC at baseline. It visually demonstrates how the addition of cMyC can reduce the intermediate “observation zone,” thereby expediting rule-in/rule-out decisions and improving emergency department patient flow. This concept is illustrated in Figure 3, showing how cMyC incorporation may reduce the observation zone compared with the ESC 0/1 h troponin algorithm.

Table 1 provides an overview of the main studies on cMyC, their findings, and limitations, which further contextualize the discussion above.

**Table 1:** Summary of key studies evaluating cardiac myosin-binding protein C (cMyC). This table summarizes the main clinical and experimental studies investigating cardiac myosin-binding protein C (cMyC) as a diagnostic biomarker in myocardial infarction. An additional column (“Study Reliability / Quality Assessment”) has been included to provide a concise evaluation of methodological robustness and the potential impact of study limitations on the validity of each conclusion, as suggested by peer review. ACS: Acute coronary syndrome; NSTEMI: Non-ST-elevation myocardial infarction; MI: Myocardial infarction; PCI: Percutaneous coronary intervention; NPV: Negative predictive value; AUC: Area under the curve; cMyC: Cardiac myosin-binding protein C; hs-TnT/hs-TnI: High-sensitivity troponin T/I; CVi/CVg: Within-subject coefficient of variation/Between-subject coefficient of variation. Data summarized from published studies [1–10]; compiled by the authors.

Author (Year)	Study Population	Setting	Main Findings	Limitations	Study Reliability/Quality Assessment
Panotopoulos et al. (2022)	120 suspected ACS patients	Single-center (Iraq)	cMyC rose and declined faster than troponins; useful for early ACS diagnosis	Small sample size; single-center	Moderate – Findings are indicative but limited by cohort size and local setting.
Kaier et al. (2015)	1,495 suspected NSTEMI patients	Large multicenter cohort (Europe)	High NPV (99.5%) for rule-out; single 0 h measurement effective	Excluded renal failure patients	High – Large cohort and robust algorithm validation enhance reliability.
Govindan et al. (2013)	MI patients vs healthy controls	Case-control	cMyC ~4.8-fold higher in MI; rapid decline post-MI	Small sample size	Moderate – Clear biomarker trend, but small-scale design limits generalizability.
Baker et al. (2015)	PCI patients (iatrogenic MI)	Interventional	cMyC detected earlier than hs-TnT; ultra-early biomarker potential	Artificial (iatrogenic MI) model	Moderate – Translational relevance demonstrated, but limited clinical applicability.
Kaier et al. (2017, APACE)	~2,000 suspected MI patients	Multicenter (Europe)	Reduced observation zone; similar AUC to hs-Tn; prognostic value	Needs external validation	High – Multicenter design and strong statistical power support high reliability.
Jacquet et al. (2009)	Cardiac tissue and plasma	Proteomic discovery	Identified cMyC as myocardium-specific biomarker	Experimental phase only	Preclinical – Foundational study; not designed for clinical inference.
Kloska et al. (2023)	Healthy adults	Cross-sectional	Established reference values and biological determinants for cMyC	Conducted in healthy volunteers only	High internal validity – Excellent assay calibration; limited generalizability.
Alaour et al. (2021)	30 healthy volunteers (10 weeks)	Serial sampling	Biological variation: CVi ≈18%, CVg ≈67%	Conducted in healthy population only	Moderate – Reliable for biological variability but limited clinical scope.
Marber et al. (2021)	Review article (expert opinion)	Narrative synthesis	Summarized translational and clinical evidence; complementary biomarker role	Not original empirical data	High scholarly reliability – Synthesizes evidence comprehensively but not experimentally.

Limitations

Despite the encouraging evidence, several limitations must be acknowledged. In larger cohorts such as the study by Kaier et al., patients with advanced renal failure or those on dialysis were excluded, limiting the applicability of results to this high-risk population. In addition, although Baker et al. provided important translational insights in the interventional setting, their analysis was based on iatrogenic myocardial infarction during PCI, which may not fully reflect spontaneous acute MI presentations.

In addition, current evidence is largely focused on short-term diagnostic accuracy and early rule-in/rule-out algorithms, whereas long-term outcome data remain limited. Furthermore, current reference values have been derived mainly from carefully selected healthy populations, as shown by Kloska et al. (2023), which may limit generalizability to real-world patients with comorbidities [8]. Moreover, although biological variation has been characterized in healthy populations (Alaour et al., 2021), it remains uncertain how these parameters perform in acute MI, renal impairment, or multi-morbidity, limiting direct clinical applicability [9]. Finally, the lack of large randomized prospective trials means that the clinical utility of cMyC has yet to be firmly established in guideline-directed practice.

Future perspectives

Looking ahead, cMyC holds promise for several important clinical applications. Large multicenter trials and randomized prospective studies are needed to validate its diagnostic thresholds and confirm its safety in real-world populations. Combining cMyC with troponins may further enhance diagnostic accuracy, as suggested by early data showing improved classification performance with dual biomarker strategies [4,7,9,10]. Because of its abundance and rapid release, cMyC may also be particularly suited for point-of-care testing, where portable assays could accelerate decision-making in resource-limited or emergency settings [8,9]. Beyond acute MI, cMyC could potentially serve as a monitoring biomarker in patients undergoing cardiac surgery, in myocarditis, or in cardio-oncology, where rapid detection of injury is essential. Finally, cost-effectiveness analyses are required to determine their impact on healthcare resource utilization, particularly in reducing unnecessary admissions and repeat testing.

## Conclusions

cMyC is a promising cardiac biomarker that may offer potential advantages over troponins due to its rapid kinetics. Limited evidence suggests potential utility for early diagnosis of myocardial infarction and for monitoring patients during hospitalization, though significant validation studies are still needed. While limitations remain, including smaller sample sizes and lack of long-term outcome data, cMyC may serve as an alternative or complement to the ESC 0/1-hour troponin algorithm. Further large-scale, multicenter studies are needed to validate its clinical role and to address issues of assay standardization, cost-effectiveness, and accessibility before widespread implementation can be achieved.
